# Supplementary material for: Frontier Orbital Engineering in Heteroatom-Doped Prototypical Organic Dyes for Dye-Sensitized Solar Cells
Source: J Phys Chem A. 2026 May 29;130(23):4331–41. doi: 10.1021/acs.jpca.6c01029 (PMC13308993; doi:10.1021/acs.jpca.6c01029)
Supplement: Supplementary file 1 [file jp6c01029_si_001.pdf]

Supporting Information for
Frontier Orbital Engineering in Heteroatom-Doped Prototypical Organic Dyes for Dye-Sensitized Solar Cells

Aditi Singh[a,†], Ram Dhari Pandey[a,†], Subrata Jana[a], Prasanjit Samal[b,†], Paweł Tecmer[a] and Szymon Śmiga[a,†]

[a]) Institute of Physics, Faculty of Physics, Astronomy and Informatics, Nicolaus Copernicus University in Toruń, Grudziadzka 5, 87-100 Toruń, Poland
[b]) School of Physical Sciences, National Institute of Science Education and Research, An OCC of Homi Bhabha National Institute, Bhubaneswar 752050, India
Email: aditisingh4812@gmail.com, pandey123iitian@gmail.com, szsmiga@fizyka.umk.pl

May 14, 2026

References

[1] Ram Dhari Pandey, Matheus Morato F. de Moraes, Katharina Boguslawski, and Pawel Tecmer, Journal of Chemical Theory and Computation, 21, 10, 5049–5061, 2025

[2] Aditi Singh, Subrata Jana, Lucian A. Constantin, Fabio Della Sala, Prasanjit Samal, and Szymon Śmiga., The Journal of Physical Chemistry Letters, 16, 32, 8198-8208, 2025

[3] Qiming Sun, et al., The Journal of Chemical Physics, 153, 024109, 2020

[4] Evgeny Epifanovsky, et al., The Journal of Chemical Physics, 155, 084801, 2021

[5] Frank Neese, WIREs Computational Molecular Science, 12, 5, e1606, 2022

1 Supplementary Tables

Table S1: Table shows the effective (ω_{eff}) and IP-tuned (ω_{IP}) range-separation parameters (in bohr^{-1}) for the fully relaxed geometries of mono-BN-doped naphthalene. The geometries were taken from Ref.[1]. The (ω_{eff}) values were computed following the methodology of Ref.[2] using PySCF [3], while the (ω_{IP}) parameters were obtained with Q-Chem [4].

Molecule	ω_{eff}	ω_{IP}
BN-1,2 naphthalene	0.263	0.244
BN-1,9 naphthalene	0.265	0.237
BN-9,1-naphthalene	0.264	0.241
BN-9,10-naphthalene	0.265	0.260

Table S2: Table shows the effective (ω_{eff}) and IP-tuned (ω_{IP}) range-separation parameters (in bohr^{-1}) for the fully relaxed geometries of mono-, di-, and tri-doped organic dyes. The (ω_{eff}) values were computed following the methodology of Ref.[2] using PySCF[3], while the (ω_{IP}) parameters were obtained with Q-Chem[4]. The geometries are optimized using ORCA[5] with BP86 functional and cc-pVDZ basis.

Molecule	ω_{eff}	ω_{IP}
NCC	0.220	0.165
CNC	0.220	0.164
CCN	0.220	0.166
OCC	0.220	0.166
COC	0.221	0.166
CCO	0.219	0.166
NNC	0.220	0.169
CNN	0.220	0.169
NCN	0.220	0.170
OOC	0.221	0.169
COO	0.221	0.171
OCO	0.219	0.171
NNN	0.220	0.161
OOO	0.221	0.174
BCN	0.220	0.161
BBO	0.219	0.148
BBN	0.219	0.148
BNN	0.220	0.165
BBB	0.219	0.143
BCC	0.220	0.157
BCO	0.219	0.160
BOO	0.220	0.165
CBC	0.219	0.158
CBN	0.219	0.165
CBO	0.219	0.164
CCC	0.220	0.162
BBC	0.220	0.163
BNC	0.219	0.147

Table S3: Table shows the ionization potential in eV using fully relaxed geometries of mono-, di-, and tri-doped organic dyes calculated using Q-Chem[4] with LC-ωPBE functional and def2-TZVPD basis set.

	Ionization Potential	
Molecule	LC-ω_{eff}PBE	LC-ω_{IP}PBE
NCC	6.735	6.430
CNC	6.683	6.370
CCN	6.778	6.468
OCC	6.863	6.555
COC	6.887	6.572
CCO	6.876	6.566
NNC	6.697	6.419
CNN	6.716	6.433
NCN	6.787	6.506
OOC	7.021	6.727
COO	7.037	6.746
OCO	7.010	6.730
NNN	6.694	6.340
OOO	7.173	6.904
BCN	6.811	6.496
BBO	6.936	6.564
BBN	6.751	6.376
BNN	6.833	6.506
BBB	7.195	6.846
BCC	6.800	6.414
BCO	6.950	6.580
BOO	7.129	6.789
CBC	6.827	6.463
CBN	6.896	6.580
CBO	6.991	6.661
CCC	6.732	6.400
BBC	6.909	6.553
BNC	6.787	6.455

Table S4: Table shows the HOMO, LUMO, and HOMO-LUMO gap in eV using fully relaxed geometries of mono-, di-, and tri-doped organic dyes calculated using Q-Chem[4] with LC-ωPBE functional and def2-TZVPD basis set.

	HOMO		LUMO		HOMO-LUMO gap	
Molecule	**LC-ω_{eff}PBE**	**LC-ω_{IP}PBE**	**LC-ω_{eff}PBE**	**LC-ω_{IP}PBE**	**LC-ω_{eff}PBE**	**LC-ω_{IP}PBE**
NCC	-6.735	-6.430	-1.505	-1.731	5.230	4.699
CNC	-6.683	-6.370	-1.486	-1.723	5.197	4.647
CCN	-6.778	-6.468	-1.380	-1.622	5.398	4.846
OCC	-6.863	-6.555	-1.663	-1.886	5.200	4.669
COC	-6.887	-6.572	-1.682	-1.913	5.205	4.659
CCO	-6.876	-6.566	-1.540	-1.774	5.336	4.792
NNC	-6.697	-6.419	-1.393	-1.600	5.304	4.819
CNN	-6.716	-6.433	-1.312	-1.529	5.404	4.904
NCN	-6.787	-6.506	-1.192	-1.407	5.595	5.099
OOC	-7.021	-6.727	-1.742	-1.951	5.279	4.776
COO	-7.037	-6.746	-1.63	-1.842	5.407	4.904
OCO	-7.010	-6.730	-1.527	-1.731	5.483	4.999
NNN	-6.749	-6.490	-1.162	-1.355	5.587	5.135
OOO	-7.173	-6.904	-1.657	-1.850	5.516	5.054
BCN	-6.811	-6.496	-2.163	-2.512	4.648	3.984
BBO	-6.936	-6.564	-3.023	-3.257	3.913	3.307
BBN	-6.751	-6.376	-2.903	-3.146	3.848	3.230
BNN	-6.833	-6.506	-1.758	-2.049	5.075	4.457
BBB	-7.195	-6.846	-3.289	-3.491	3.906	3.355
BCC	-6.800	-6.414	-2.240	-2.612	4.560	3.802
BCO	-6.950	-6.580	-2.264	-2.615	4.686	3.965
BOO	-7.129	-6.789	-2.161	-2.471	4.968	4.318
CBC	-6.827	-6.463	-2.300	-2.642	4.527	3.821
CBN	-6.896	-6.580	-1.780	-2.087	5.116	4.493
CBO	-6.991	-6.661	-2.046	-2.367	4.945	4.294
CCC	-6.732	-6.400	-1.657	-1.905	5.075	4.495
BBC	-6.909	-6.553	-3.029	-3.255	3.880	3.298
BNC	-6.787	-6.455	-1.807	-2.123	4.980	4.332

Table S5: Table shows singlet-singlet (SS) and singlet-triplet (ST) excitation energies in eV using fully relaxed geometries of mono-, di-, and tri-doped organic dyes calculated using Q-Chem[4] with Tamm-Dancoff Approximation (TDA) on LC-ωPBE functional and def2-TZVPD basis set.

	SS		ST	
**Molecule**	**LC-**$\omega_{eff}PBE$	**LC-**$\omega_{IP}PBE$	**LC-**$\omega_{eff}PBE$	**LC-**$\omega_{IP}PBE$
NCC	2.61	2.57	1.28	1.26
CNC	2.64	2.59	1.31	1.29
CCN	2.72	2.64	1.46	1.43
OCC	2.60	2.54	1.26	1.24
COC	2.63	2.57	1.28	1.26
CCO	2.67	2.59	1.39	1.36
NNC	2.73	2.69	1.38	1.37
CNN	2.79	2.71	1.54	1.50
NCN	2.95	2.88	1.68	1.65
OOC	2.72	2.66	1.36	1.34
COO	2.78	2.70	1.49	1.46
OCO	2.85	2.77	1.58	1.55
NNN	2.98	2.91	1.69	1.67
OOO	2.92	2.84	1.62	1.59
BCN	1.36	1.20	0.53	0.46
BBO	0.99	0.98	-0.07	0.02
BBN	0.92	0.89	-0.17	-0.07
BNN	1.76	1.64	0.98	0.93
BBB	1.34	1.39	0.09	0.23
BCC	1.30	1.13	0.45	0.38
BCO	1.36	1.20	0.53	0.46
BOO	1.63	1.49	0.84	0.77
CBC	1.21	1.08	0.39	0.33
CBN	1.73	1.58	0.98	0.91
CBO	1.54	1.40	0.78	0.71
CCC	2.46	2.41	1.12	1.10
BBC	1.09	1.10	-0.03	0.07
BNC	1.69	1.53	0.93	0.87

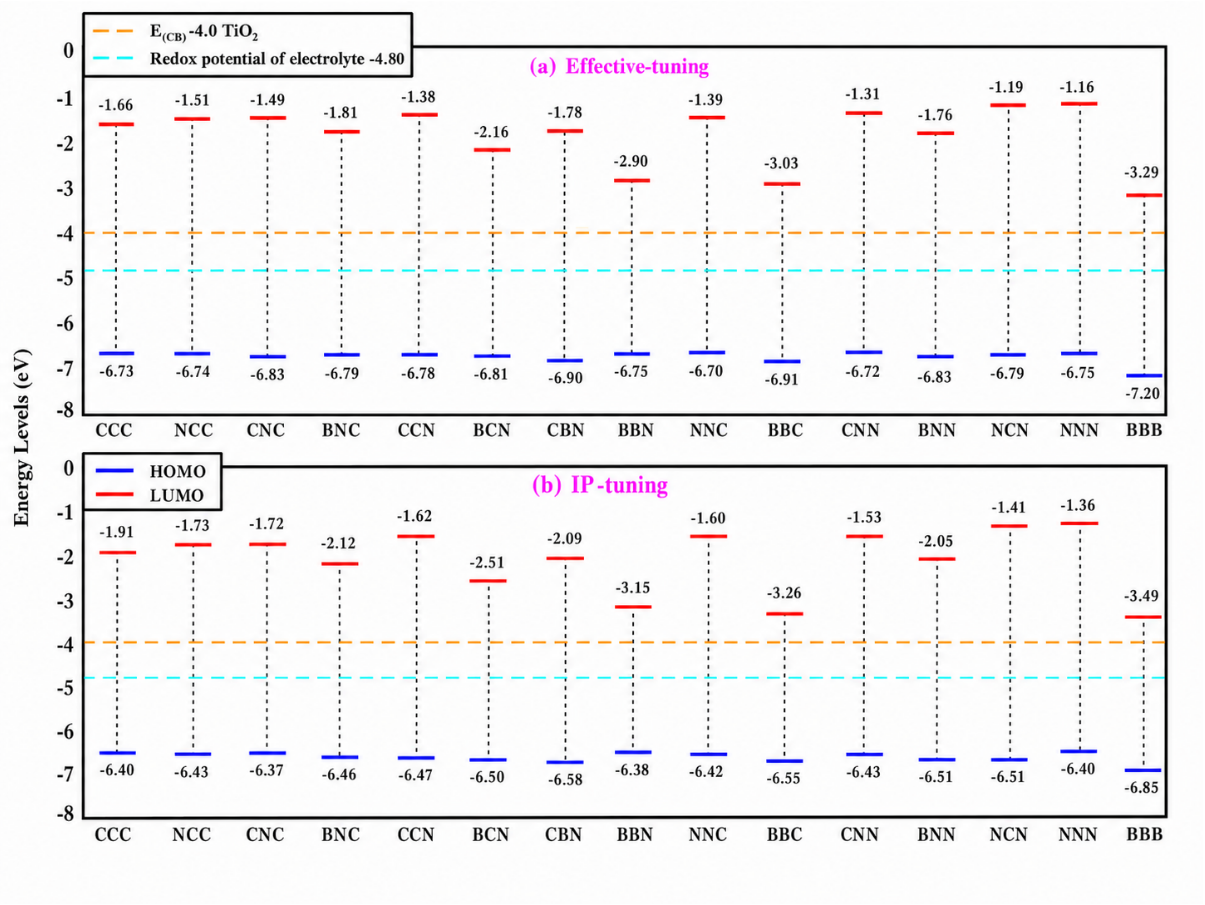

Figure S1: Illustrates the HOMO and LUMO energy levels (eV) of nitrogen- and boron-doped organic dyes, analyzing the performance of the LC-ωPBE functional using the two tuning variants: effective (ω_{eff}) and IP tuning (ω_{IP}). The complete data is available in Table S4.

2 Supplementary Figures

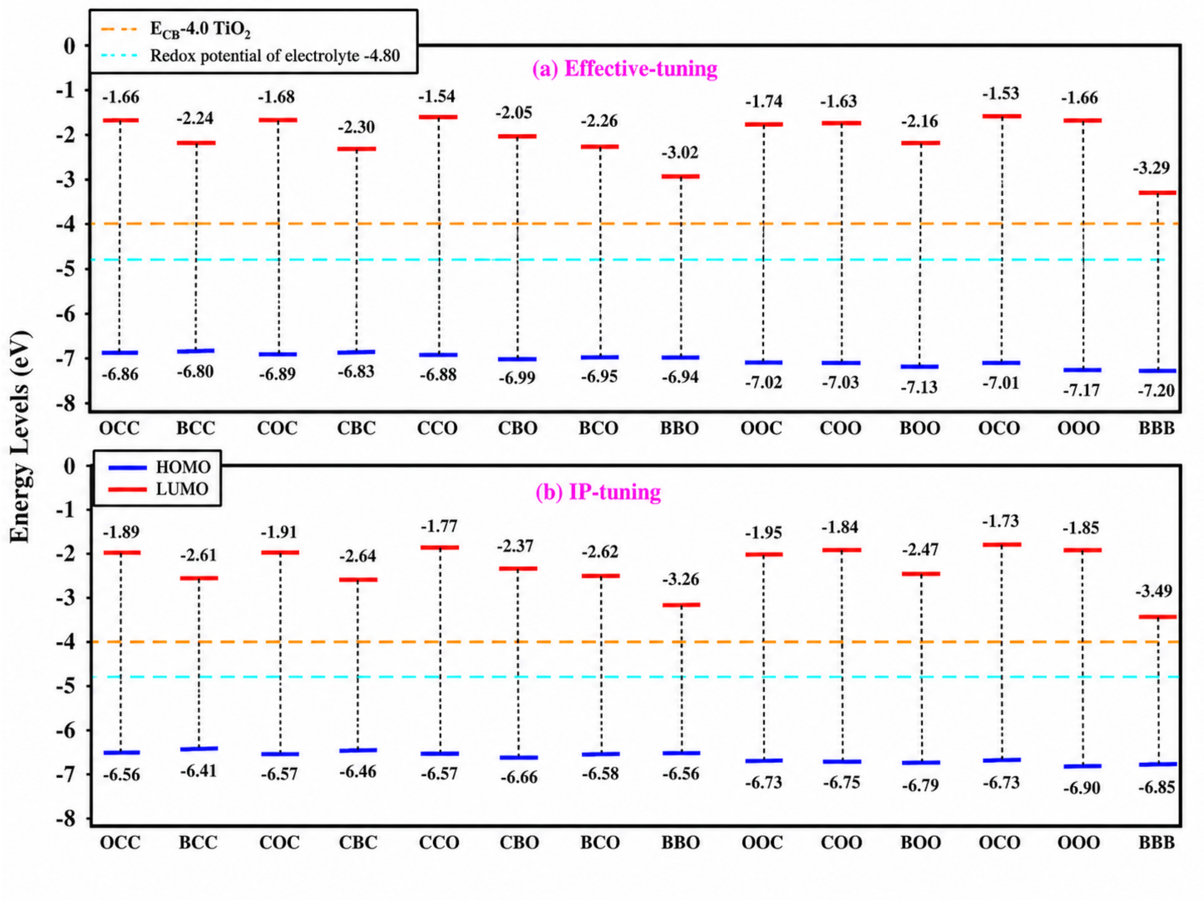

Figure S2: Illustrates the HOMO and LUMO energy levels (eV) of oxygen- and boron-doped organic dyes, analyzing the performance of the LC-ωPBE functional using the two tuning variants: effective (ω_{eff}) and IP tuning (ω_{IP}). The complete data is available in Table S4.

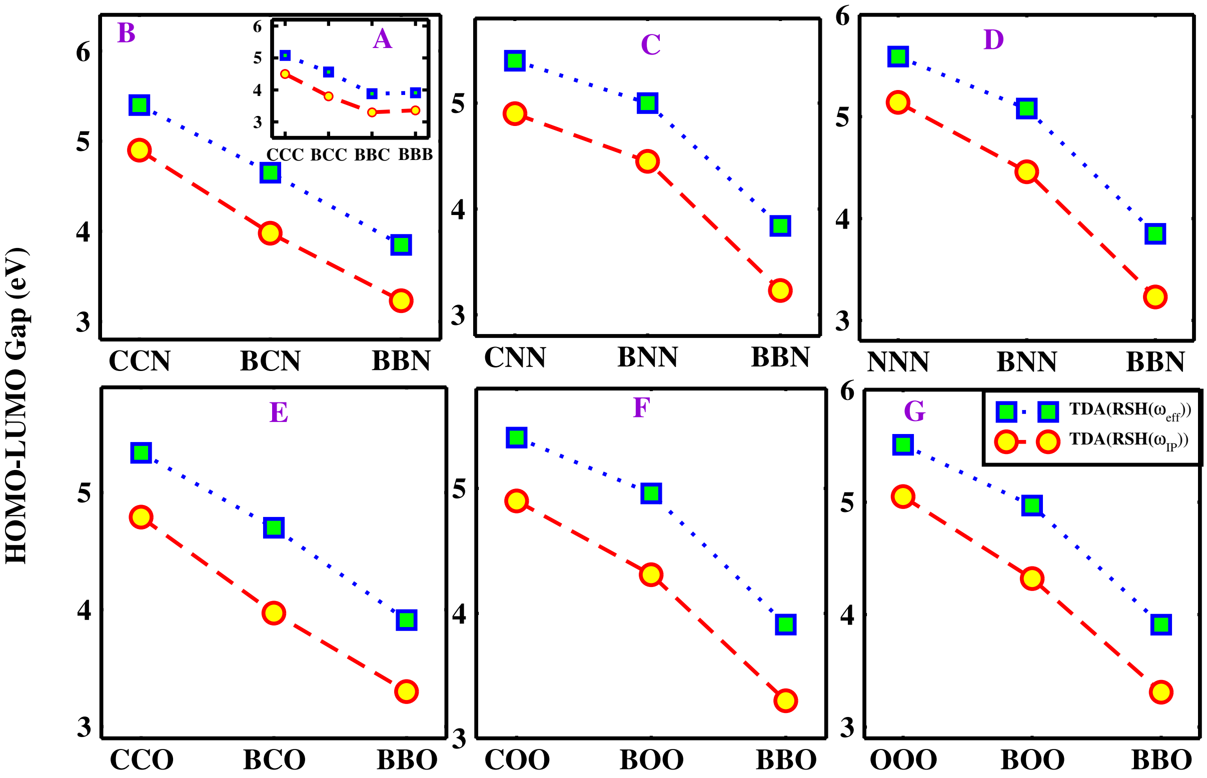

Figure S3: Illustrates the HOMO-LUMO gap trends for the doped system, analyzing the performance of the LC-ωPBE functional using the two tuning variants: effective (ω_{eff}) and IP tuning (ω_{IP}). The complete data is available in Table S4.

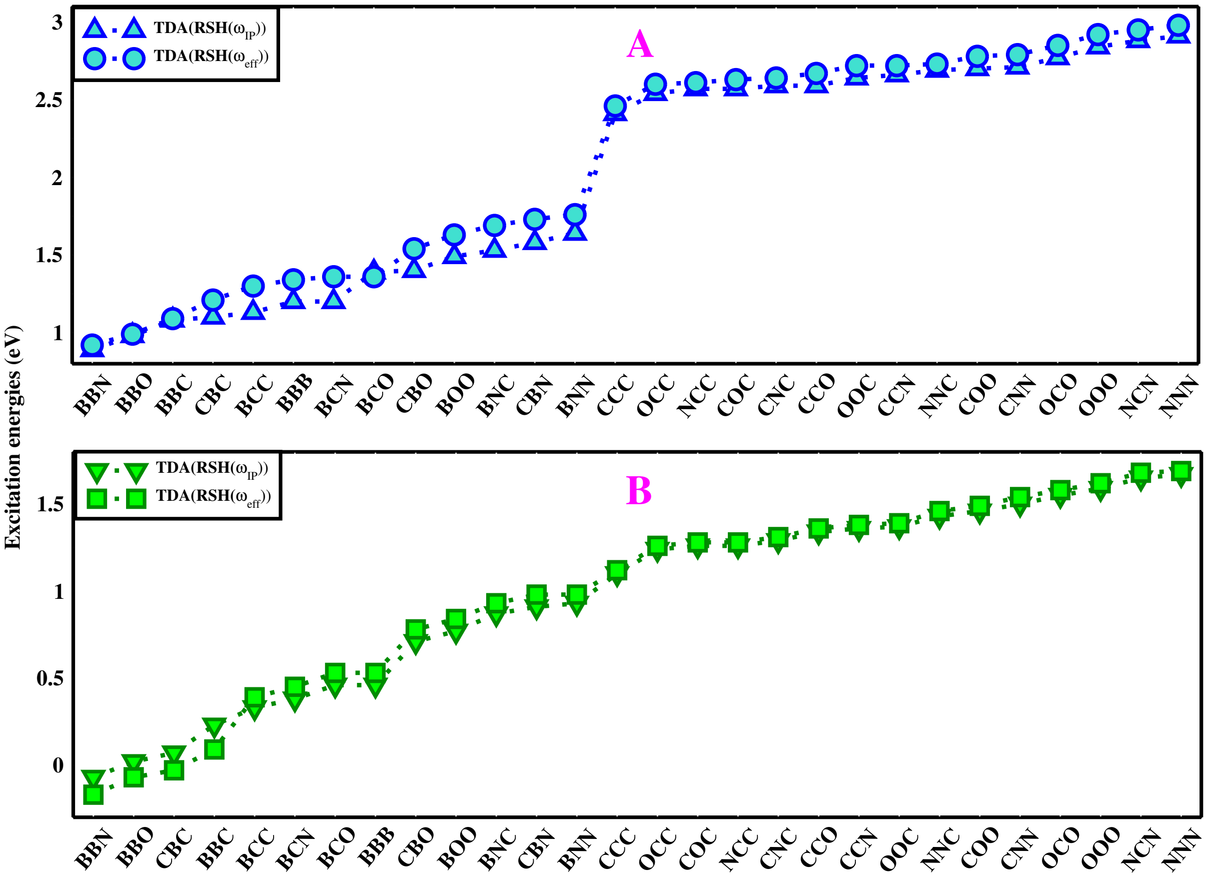

Figure S4: Illustrates the singlet-singlet excitation energies (eV)(A) and singlet-triplet excitation energies (eV)(B) of nitrogen-, oxygen-, and boron-doped organic dyes, analyzing the performance of the LC-ωPBE functional using the two tuning variants: effective (ω_{eff}) and IP tuning (ω_{IP}). The complete data is available in Table S5.
